# Treatment of Fistula in Ano—A New Method

**Published:** 1879-03-20

**Authors:** I. J. Suggs

**Affiliations:** Texas


### *TREATMENT OF FISTULA IN ANO.—A NEW METHOD.*

BY I. J. SUGGS, M.D., OF TEXAS.

Some years since I was called in consultation in case of fistula in ano. After examining the case, I conceived the idea of introducing a tube into the rectum so as to prevent the action of the sphincter, and thereby allowing the fistula to heal. It was agreed to. I had a silver tube made as follows: 3 inches long, about 1½ inches in diameter at the point, larger at the base, flared some small holes in the base so as to confine it with a bandage. Made an obturator similar to that of a speculum uteri. After having the bowels well evacuated the tube was introduced and confined with a T bandage, the obturator removed and a plug of cotton introduced, the fistula well cleansed and a diluted tincture of iodine injected. Five days after the operation the tube was removed and the fistula perfectly cured.

I have not had a case of fistula since in which to try again the above treatment.

